# Adaptation of *Pseudomonas aeruginosa* biofilms to tobramycin and the quorum sensing inhibitor C-30 during experimental evolution requires multiple genotypic and phenotypic changes

**DOI:** 10.1099/mic.0.001278

**Published:** 2023-01-18

**Authors:** Mona Bové, Mette Kolpen, Mads Lichtenberg, Thomas Bjarnsholt, Tom Coenye

**Affiliations:** ^1^​ Laboratory of Pharmaceutical Microbiology, Ghent University, Ghent, Belgium; ^2^​ Department of Clinical Microbiology, Rigshospitalet, 2200 Copenhagen N, Denmark; ^3^​ Costerton Biofilm Center, Department of Immunology and Microbiology, University of Copenhagen, Copenhagen, Denmark

**Keywords:** antimicrobial resistance, experimental evolution, *Pseudomonas aeruginosa*

## Abstract

In the present study we evaluated the fitness, antimicrobial susceptibility, metabolic activity, gene expression, *in vitro* production of virulence factors and *in vivo* virulence of experimentally evolved *Pseudomonas aeruginosa* PAO1. These strains were previously evolved in the presence of tobramycin and the quorum sensing inhibitor furanone C-30 (C-30) and carried mutations in *mexT* and *fusA1*. Compared to the wild-type (WT), the evolved strains show a different growth rate and different metabolic activity, suggesting they have an altered fitness. *mexT* mutants were less susceptible to C-30 than WT strains; they also show reduced susceptibility to chloramphenicol and ciprofloxacin, two substrates of the MexEF-OprN efflux pump. *fusA1* mutants had a decreased susceptibility to aminoglycoside antibiotics, and an increased susceptibility to chloramphenicol. The decreased antimicrobial susceptibility and decreased susceptibility to C-30 was accompanied by a changed metabolic activity profile during treatment. The expression of *mexE* was significantly increased in *mexT* mutants and induced by C-30, suggesting that MexEF-OprN exports C-30 out of the bacterial cell. The *in vitro* production of virulence factors as well as virulence in two *in vivo* models of the strains evolved in the presence of C-30 was unchanged compared to the virulence of the WT. Finally, the evolved strains were less susceptible towards tobramycin (alone and combined with C-30) in an *in vivo* mouse model. In conclusion, this study shows that mutations acquired during experimental evolution of *P. aeruginosa* biofilms in the presence of tobramycin and C-30, are accompanied by an altered fitness, metabolism, *mexE* expression and *in vitro* and *in vivo* antimicrobial susceptibility.

## Introduction

Infections with *Pseudomonas aeruginosa* are difficult to treat due to acquired and intrinsic resistance to many antibiotics [1]. *P. aeruginosa* can acquire resistance via horizontal gene transfer or through mutations, while intrinsic resistance can be the result of a reduced outer membrane permeability or increased efflux [2]. The resistance-nodulation-cell division (RND) transporter family plays a major role in multidrug resistance in *P. aeruginosa *, with MexAB-OprM, MexCD-OprJ, MexEF-Oprn and MexXY being its most important multidrug efflux pumps [3]. The MexEF-OprN efflux pump consists of a membrane fusion protein (MexE), a transmembrane transporter (MexF) and an outer membrane factor (OprN) [4]. Basal expression levels of the *mexEF-oprN* genes are low in most *P. aeruginosa* strains and increased expression is linked to resistance to fluoroquinolones, chloramphenicol, imipenem and trimethoprim [5, 6]. This phenotype was first observed in strains that were spontaneously resistant to norfloxacin (*nfxC* mutants) [7]. Subsequently it was discovered that mutations in several genes result in an increased *mexEF-oprN* expression (explaining the phenotypes observed in *nfxC* mutants), including mutations in the genes encoding the transcriptional activator MexT and the oxidoreductase MexS [8–10]. *mexT* and *mexS* are located in close proximity of the MexEF-OprN operon, and MexT and MexS are thought to counteract each other, with MexT stimulating the expression of *mexEF-oprN,* while MexS has an inhibitory role. In addition, MexT activates the expression of *mexS* [11]. Strains harbouring *mexT* mutations also have a reduced *oprD* expression [12]. In *P. aeruginosa,* the outer membrane protein OprD is responsible for basal resistance to carbapenems (in particular imipenem), explaining why strains with mutations in *mexT* show increased imipenem resistance [13].

The production of many virulence factors as well as biofilm formation and antimicrobial susceptibility of biofilms are (at least partly) controlled by quorum sensing (QS). Because of this, inhibition of one or more bacterial QS systems has the potential to decrease virulence and increase susceptibility to antimicrobial therapies [14–21]. QS inhibition does not rely on inhibiting growth and/or killing of micro-organisms, and it is generally assumed that it will result in less selective pressure and resistance development compared to conventional antimicrobial compounds, although evidence for the latter statement is scarce [22, 23]. The brominated furanone C-30 (C-30), isolated from the marine alga *Delisea pulchra*, is an example of such a QS inhibitor [24]. While C-30 has strong QS inhibitory activity in *P. aeruginosa* [25, 26] and potentiates the activity of antibiotics against *P. aeruginosa* biofilms [17], it was shown that exposure to C-30 can select for mutations in repressors of the MexAB-OprM efflux pump (including *mexR* and *nalD*) and that resistance due to reduced permeability is observed in clinical *P. aeruginosa* isolates [27–29]. In addition, results from an experimental evolution study in which *P. aeruginosa* PAO1 was experimentally evolved in a synthetic cystic fibrosis medium (SCFM2) in the presence of tobramycin and/or C-30, showed that C-30 rapidly loses its tobramycin-potentiating activity against *P. aeruginosa* biofilms [30]. Mutations in *mexT* and *fusA1* were acquired in all lineages exposed to C-30 and tobramycin, respectively [30]. The 8 bp deletion that was detected in *mexT* removes a premature stop codon allowing expression of a full-length *mexT* [31]. FusA1 on the other hand is a part of elongation factor G, which is essential for the elongation and recycling step during translation [32]. Aminoglycoside antibiotics interfere with translation and point mutations in *fusA1* are frequently detected in aminoglycoside resistant *P. aeruginosa* strains [33]. In addition, mutations in *fusA1* were also observed in another study in which *P. aeruginosa* was exposed to the combination tobramycin +C-30 [34].

Mutations like the deletion in *mexT* that are beneficial in the presence of the selective pressure are often disadvantageous in the absence of that pressure, i.e. evolved strains show reduced fitness in the absence of the selective pressure. This evolutionary ‘tradeoff’ might be reflected in a reduced growth rate or a decreased virulence [35, 36]. In the present study we evaluated the effect of the mutations that were acquired during the experimental evolution on fitness, antimicrobial susceptibility (*in vitro* and *in vivo*), metabolic activity and virulence of *P. aeruginosa *. Additionally, the effect of the *mexT* deletion (present in all lineages that were exposed to C-30) on the expression of *mexE, mexS, mexT* and *oprD* was investigated.

## Methods

### Strains and culture conditions

The strains used in this study were obtained by experimental evolution as previously described [30] (Tables S1 and S2, available with the online version of this article). Briefly, *P. aeruginosa* PAO1 biofilms were repeatedly exposed to the QSI C-30 (100 µg ml^−1^), tobramycin (20 µg ml^−1^), or a combination of C-30 and tobramycin, in SCFM2, during 16 cycles. *P. aeruginosa* was maintained on tryptone soy agar (TSA, Neogen) and all overnight cultures were prepared in Lysogeny Broth (LB, Neogen). For each condition three independent replicate populations (lineages) were used. Whole populations of the evolved lineages were used for subsequent analyses.

### Determination of minimum inhibitory concentration

The MIC of amikacin, gentamicin, ciprofloxacin, chloramphenicol and imipenem (all obtained from Sigma), and tobramycin (obtained from TCI Europe) was determined according to the EUCAST guidelines [37]. Briefly, twofold serial dilutions of antibiotics were made in Mueller–Hinton broth (Neogen) and approx. 10^5^ c.f.u. ml^−1^ *P. aeruginosa* was added to the wells of a flat bottom 96-well microtitre plate (total volume of 200 µl/well). After 24 h growth at 37 °C, the optical density (OD)_590_ was measured with an Envision multimode plate reader (PerkinElmer) and the MIC was defined as the antibiotic concentration that fully inhibited the growth. MICs were determined for the WT *P. aeruginosa* PAO1 and the lineages that were experimentally evolved in the presence of tobramycin, C-30 or the combination of both (three lineages for each treatment, the median MIC for the three lineages was calculated).

### Determination of minimum bactericidal concentration

The entire content of the wells with an antibiotic concentration higher than the MIC (i.e. all wells that showed no visible growth after 24 h) was plated on LB plates. The minimum bactericidal concentration (MBC) was defined as the lowest concentration of the antibiotic that resulted in complete absence of growth after 24 h incubation at 37 °C.

### Bacterial growth curves

Overnight cultures of *P. aeruginosa* were diluted in LB broth to obtain approx. 5×10^5^ c.f.u. ml^−1^. C-30 (Sigma) was added in a final concentration range of 2–16 µg ml^−1^ and 200 µl of the mixture was incubated in a round bottom 96-well plate at 37 °C. The OD_600_ was measured during 24 h every 30 min with a VICTOR Nivo Multimode Plate Reader (PerkinElmer). For the calculation of the duration of the lag phase and growth rate, the growth data was fitted to a Gompertz model using the SigmaPlot software (version 14.5, Systat Software).

### Determination of metabolic activity using isothermal microcalorimetry

Isothermal microcalorimetry (IMC) was performed with the calScreener device (Symcel), according to the manufacturer’s instructions. Overnight cultures of the WT and evolved *P. aeruginosa* PAO1 were diluted in LB broth to a final concentration of 2.5×10^7^ c.f.u. ml^−1^ in all IMC experiments. Antibiotics were added to obtain a final concentration equal to 0.5× (gentamicin, ciprofloxacin and chloramphenicol) or 0.25× (tobramycin and amikacin) the MIC for the WT strain. C-30 was added to obtain a final concentration of 2, 4, 8 or 16 µg ml^−1^. Thermograms and accumulated heat were exported as .csv files (one data point every 10 min) and principal component analysis (PCA) of raw heat flow values [38] was subsequently performed using ClustVis [39]. Based on the thermograms, time to peak (the time point at which the maximum heat flow is reached), maximum metabolic rate (the maximum heat flow or maximum metabolic activity) and maximum metabolic velocity (maximum value of the first derivative of the heat flow, i.e. the maximum speed at which the heat flow is increasing) were calculated using the calView software (Symcel).

### RT-PCR evaluation of *mexE* expression

Overnight cultures of *P. aeruginosa* were diluted to 1×10^7^ c.f.u. ml^−1^ (OD_590_=0.01) in 20 ml of LB broth and cultivated in a shaking water bath (250 r.p.m., 37 °C). Cells were harvested at mid exponential phase (OD_590_=0.5). To evaluate if the expression of *mexEF-oprN* requires exposure to C-30, the compound (2 µg ml^−1^) was added to the bacteria 30 min prior to harvesting. The Ribopure RNA purification kit for bacteria (Invitrogen) was used to extract RNA of about 4×10^9^ cells, after which the samples were treated with DNAase for 30 min to remove traces of genomic DNA. The RNA concentration after extraction was determined using a BioDrop µLITE (BioDrop). Next, the High-Capacity cDNA Reverse Transcription kit (Applied Biosystems) was used to convert 500 ng of RNA to cDNA. The RT-PCR reactions were run on a CFX96 Real-Time System C1000 Thermal Cycler (Bio-Rad) and the protocol consisted of 3 min at 95 °C followed by 50 amplification cycles consisting of 15 s at 95 °C, 30 s at 64 °C and 15 s at 72 °C. The reaction mix included 10 µl of the GoTaq qPCR mastermix (Promega), 0.6 µl primer mix (10 µM), 2 µl of cDNA and 7.4 µl of RNAase free water. Primers used were GCGGGTGTCGGGCTACATC (forward) and CGGCGTCGAAGTAGGCGTAG (reverse) for *mexE* [10]; TATTGATGCCGAACCTGCTG (forward) and GGAGGATCTTCGGCTTGCTG (reverse) for *mexT*, AGGGCGTCAATGTCATCCTC (forward) and CTGCAGGTGCTTCTTGAACG (reverse) for *mexS*, ATTGCACTGGCGGTTTCC (forward) and ATGAACCCCTTCGCTTCG (reverse) for *oprD* [40]; *rpsL* was used as a reference gene (forward: GCAAGCGCATGGTCGACAAGA, reverse: CGCTGTGCTCTTGCAGGTTGTGA) [41]. Melt curve analysis and no-RT controls were included to confirm that there was a unique PCR product and to confirm the absence of residual gDNA, respectively. Quantification cycle (Cq) values were obtained using the Bio-Rad CFX Manager 3.1 software with a manually defined baseline of 100, and the expression of the target genes in the evolved lineages was compared to the expression in the WT *P. aeruginosa* PAO1 strain with the ΔΔCq method. The expression of the reference gene *rpsL* was stable across all samples.

### Pyoverdine and pyocyanin quantification

Overnight cultures of *P. aeruginosa* were spun down (5000 r.p.m., 5 min) and resuspended in 10 ml of LB to a density of 2.5×10^7^ c.f.u. ml^−1^. After 24 h growth in a shaking water bath (100 r.p.m., 37 °C) the cultures were vortexed for 1 min and then filtered (0.22 µm, PES, Merck Millipore). Pyoverdine was quantified by measuring the absorbance at 405 nm of 200 µl supernatant with the Envision plate reader. LB broth was used as blank control [42]. To extract pyocyanin, 3.3 ml of filtered supernatant was added to 2 ml of chloroform. This mixture was vortexed for 1 min and centrifuged (5000 r.p.m., 5 min), to obtain two separate phases. Subsequently, 1 ml of the bottom chloroform phase was added to 1 ml of 0.2 M HCl. The vortex and centrifugation steps were repeated to obtain a pink coloured, aqueous top layer and the absorbance of 200 µl of this aqueous layer was measured at 520 nm^43^. Then, 0.2 M HCl was used as the blank control.

### Quantification of protease activity

Protease activity was quantified using an azocasein assay as previously described [43, 44]. Briefly, 400 µl of filtered supernatant was mixed with 400 µl of azocasein substrate (Sigma, 5 mg ml^−1^ in 0.1 M pH 8 Tris-HCL buffer) and incubated for 1 h in a shaking water bath (100 r.p.m., 37 °C). After 1 h the undegraded azocasein was precipitated by adding 100 µl of trichloroacetic acid (10%) and the mixture was spun down. Subsequently 100 µl of supernatant was added to 100 µl of NaOH (625 mM) in a 96-well plate and the absorbance was measured at 420 nm.

### Swarming motility

Swarming motility assays were performed on minimal medium (M8) plates with an agar concentration of 0.6 % (w/v) [45]. To assess the swarming motility 2 µl of a *P. aeruginosa* culture containing approx. 1×10^9^ c.f.u. ml^−1^ was put on the centre of the agar. The plates were incubated at 37 °C during 24 h after which the diameter was measured.

### Rhamnolipid production

Rhamnolipid production was quantified using methylene blue agar plates [42]. A petri dish (10 cm diameter) was filled with exactly 15 ml of methylene blue agar, after which the plates were dried for 15 min in a laminar airflow (LAF) cabinet. Subsequently a hole was punctured in the centre of the agar (using a sterile 1 ml tip) and 70 µl of 3×10^9^ c.f.u. ml^−1^ *P *. *aeruginosa* was added. The diameter of the blue halo was measured after 48 h of incubation at 37 °C.

### *Caenorhabditis elegans* infection assay

*C. elegans* (glp-4; sek-1) was cultured, harvested and infected under standard conditions, as previously described [46, 47]. Briefly, 20 nematodes were infected with 5×10^8^ c.f.u. ml^−1^ *P *. *aeruginosa* in a final volume of 100 µl in a well of a flat-bottom 96-well plate. The nematodes were incubated at 25 °C during infection, and the survival of the nematodes was quantified every 24 h during 3 days, using an EVOS FL Auto Cell Imaging system (Thermo Fischer Scientific). Nonmobile nematodes with a straight shape were scored as dead. An uninfected, untreated control group of nematodes was included in all experiments.

### Peritonitis/sepsis mouse model

The *in vivo* murine peritonitis/sepsis model was set up as previously described [48]. Briefly, eight NMRI mice per treatment group received an intraperitoneal injection with 5×10^6^ c.f.u. of *P. aeruginosa* PAO1. One hour after infection the mice were treated with 30 mg kg^−1^ BW tobramycin, 1 mg kg^−1^ BW C-30 or a combination of both, and an untreated control group was treated with saline. The clinical condition of the mice was scored with a score from 0 to 6 (0=unaffected, 1=slightly affected, 2=affected, 3=clearly affected, 4=very affected and mouse must be sacrificed, 5=motionless and cold, 6=dead) as previously described [48]. The number of c.f.u. in the blood and peritoneal fluid 2 and 4 h after treatment was determined via serial dilution and drop plating on modified Conradi-Drigalski agar (10 g l^−1^ detergent, 1 g l^−1^ Na_2_S_2_O_3_ H_2_O, 0.1 g l^−1^ bromothymol blue, 9 g l^−1^ lactose and 0.4 g l^−1^ glucose, pH8.0; SSI, Denmark).

### Statistical analysis

Statistical analysis was performed with the SPSS software 27.0. The normality of the data was checked with the Shapiro–Wilk test. A one-way ANOVA with Dunnett correction was used to compare the means of multiple groups to one control group. If normality could not be assumed, a Kruskal–Wallis one-way ANOVA test was used to compare multiple groups. When comparing the mean of two groups, we used independent samples *T*-tests.

## Results and discussion

### Lineages Evolved in the Presence of Tobramycin and C-30 Show Decreased Fitness

**Fig. 1.**
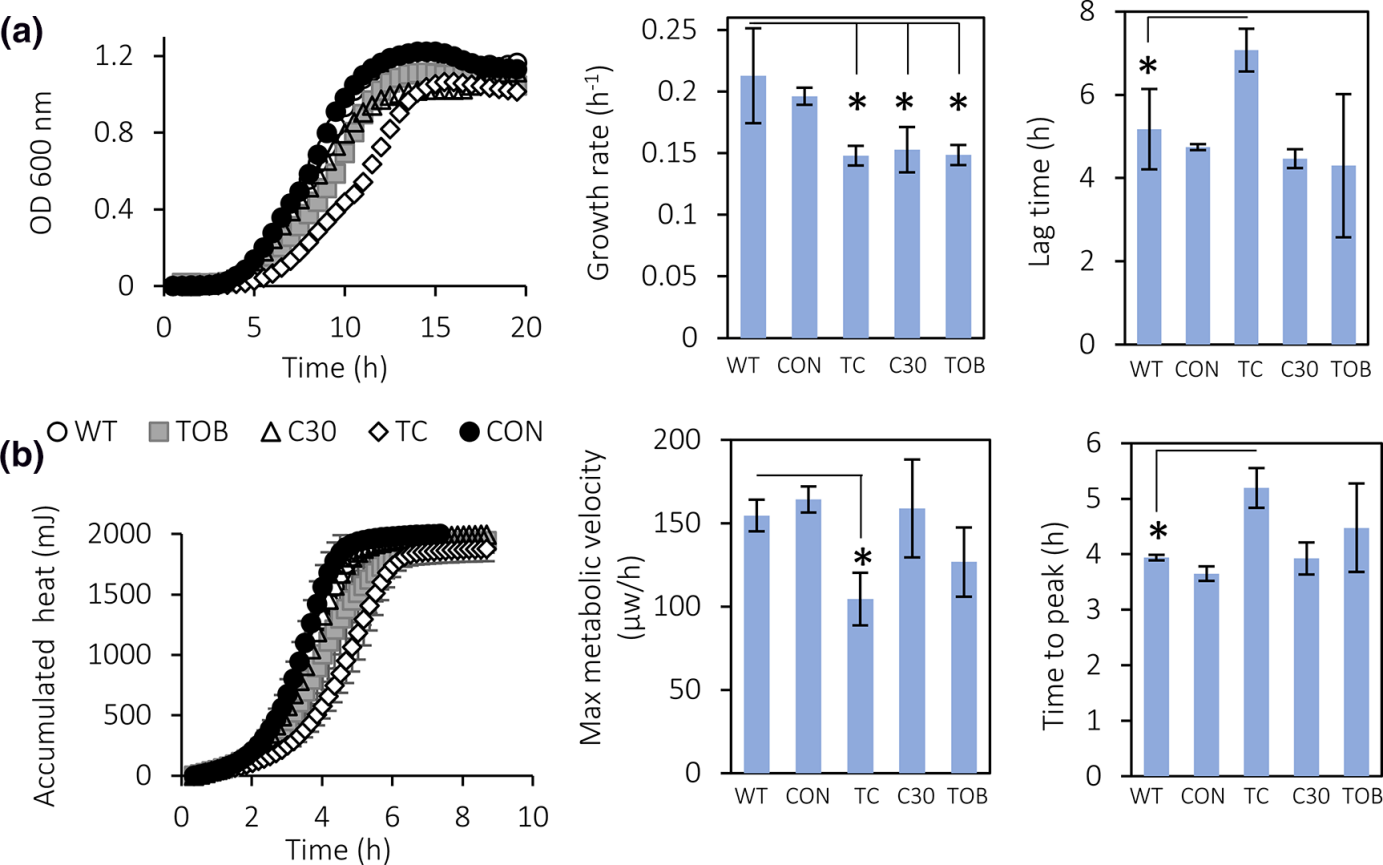
(a) Growth curve (left), growth rate during exponential phase (centre) and lag time (right) of the different *P. aeruginosa* strains investigated. (b) Total accumulated heat (left), maximum metabolic velocity (centre) and time to maximum metabolic activity (right) of the different *P. aeruginosa* strains investigated. WT: wild-type, CON: lineage evolved in the absence of antimicrobial treatment, C30: lineage evolved in the presence of C-30, TC: lineage evolved in the presence of the combination of tobramycin and C-30, TOB: lineage evolved in the presence of tobramycin. Data shown are average, error bars indicate standard deviations (*n*=3). *, significantly different from the WT (*P*<0.05). Growth curves with error bars are shown in Fig. S2.

To determine whether experimental evolution as such (i.e. in the absence of tobramycin and/or C-30) had an impact on fitness of *P. aeruginosa *, growth (Fig. 1a) and metabolic activity (Fig. 1b) of the evolved strains studied (Table S2) was compared to those of the WT *P. aeruginosa *. The growth curves and accumulated heat of the WT and the untreated evolved control were virtually identical, suggesting that prolonged growth in SCFM2 does not lead to mutations affecting growth or metabolism of *P. aeruginosa *. In addition, no significant differences were observed in lag time, time to maximum metabolic activity, maximum metabolic rate or maximum metabolic velocity between WT and strains evolved in presence of either C-30 or tobramycin (Fig. 1a and b). However, the growth rate of the lineages evolved in the presence of tobramycin, C-30 and the combination was significantly decreased (*P*<0.05) compared to the growth rate of the WT. The maximum metabolic velocity of *P. aeruginosa* lineages evolved in the presence of the combination of tobramycin and C-30 were significantly decreased (*P*<0.05), and the lag time and time to maximum metabolic activity were significantly increased (*P*<0.05). The maximum metabolic rate did not differ between WT and evolved strains (Fig. S1). Combined, these data suggest there is a fitness cost associated with the genetic changes that occur during adaptation to the combination of C-30 and tobramycin.

**Fig. 2.**
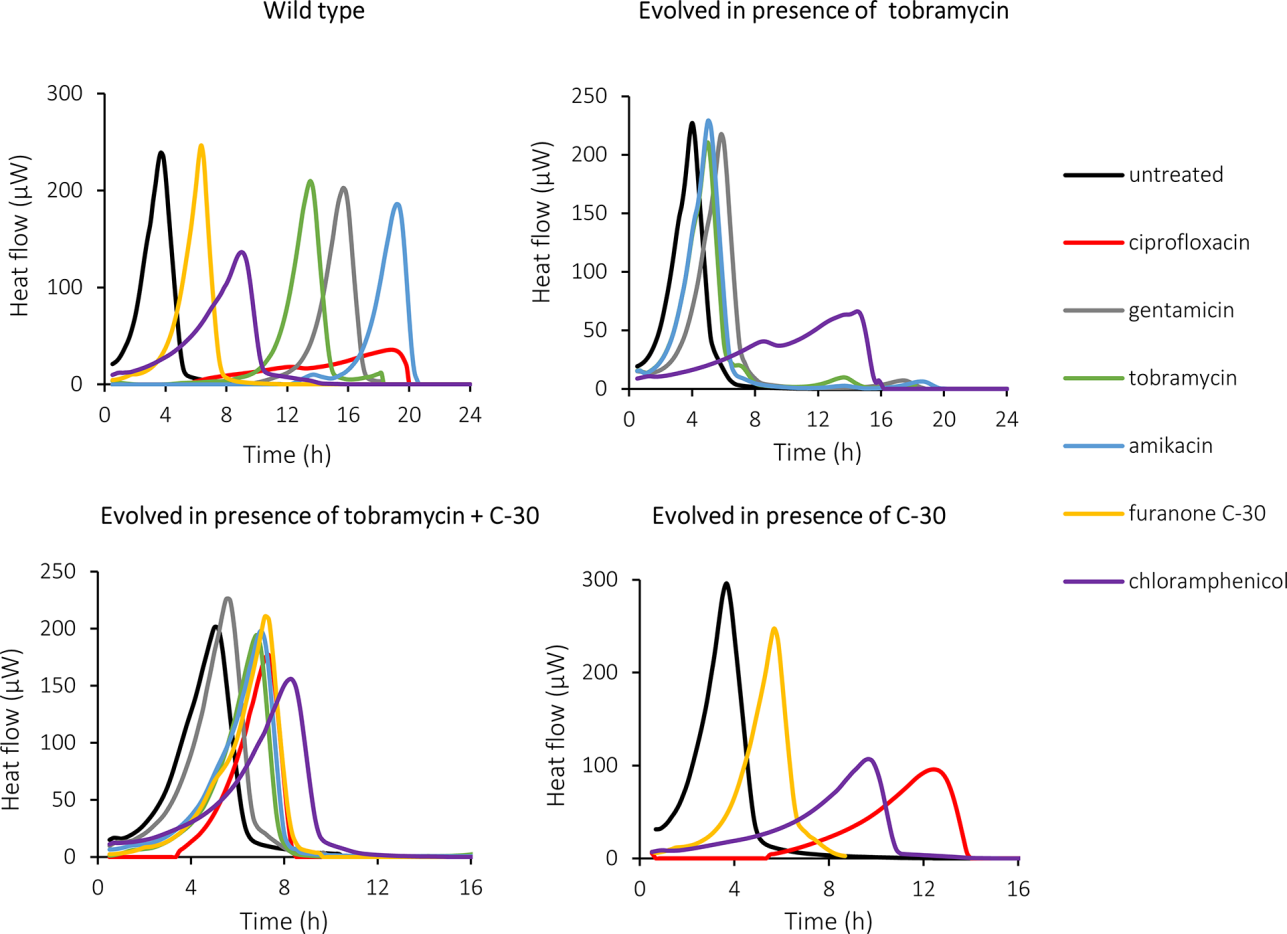
Representative thermograms of WT and evolved *P. aeruginosa* lineages that were either not treated or treated with ciprofloxacin (0.125 µg ml^−1^), gentamicin (1 µg ml^−1^), tobramycin (0.25 µg ml^−1^), amikacin (1 µg ml^−1^), C-30 (16 µg ml^−1^) or chloramphenicol (16 µg ml^−1^).

### The Evolved Lineages Show Decreased Susceptibility and Altered Metabolic Activity in Presence of MexEF-OprN Substrates and Aminoglycosides

As several lineages had acquired a mutation in *fusA1*, known to be involved in aminoglycoside resistance [32, 34, 49], the antimicrobial susceptibility towards several aminoglycoside antibiotics (tobramycin, amikacin and gentamicin) was determined. In addition, to investigate the effect of the 8 bp deletion in *mexT* on the function of the MexEF-OprN efflux pump, the MIC and MBC of several substrates of MexEF-OprN (ciprofloxacin and chloramphenicol) was determined for the WT and evolved strains. Furthermore, the influence of the aforementioned antibiotics on the metabolic activity of the evolved and WT *P. aeruginosa* was investigated with isothermal microcalorimetry.

Previous studies indicated that metabolism plays an important role in antibiotic resistance and tolerance [50–52], as for example mutations in metabolism related genes conferred antibiotic resistance in clinical *Escherichia coli* strains [53], and the activity of bactericidal antibiotics (such as aminoglycosides) is based on the production of reactive oxygen species during aerobic respiration [54]. In isothermal microcalorimetry the heat produced by bacterial metabolism is continuously measured (Fig. 2), and the resulting thermograms can be used to cluster samples [38, 55]. In addition, the maximum metabolic activity, maximum metabolic velocity and time to maximum metabolic activity can be derived from the thermogram and can be used to compare metabolism [56] . The metabolic activity of *P. aeruginosa* is affected in a different way by antibiotics of different classes; e.g. aminoglycoside antibiotics strongly delay the time to maximum metabolic activity but only have a small effect on the maximum metabolic activity, while ciprofloxacin and chloramphenicol have a pronounced impact on the maximum metabolic activity (Fig. 2). We also observed that thermograms of the evolved and WT bacteria were affected differently by the antimicrobial treatments (Fig. 2).

### Response to Aminoglycoside Treatment

**Fig. 3.**
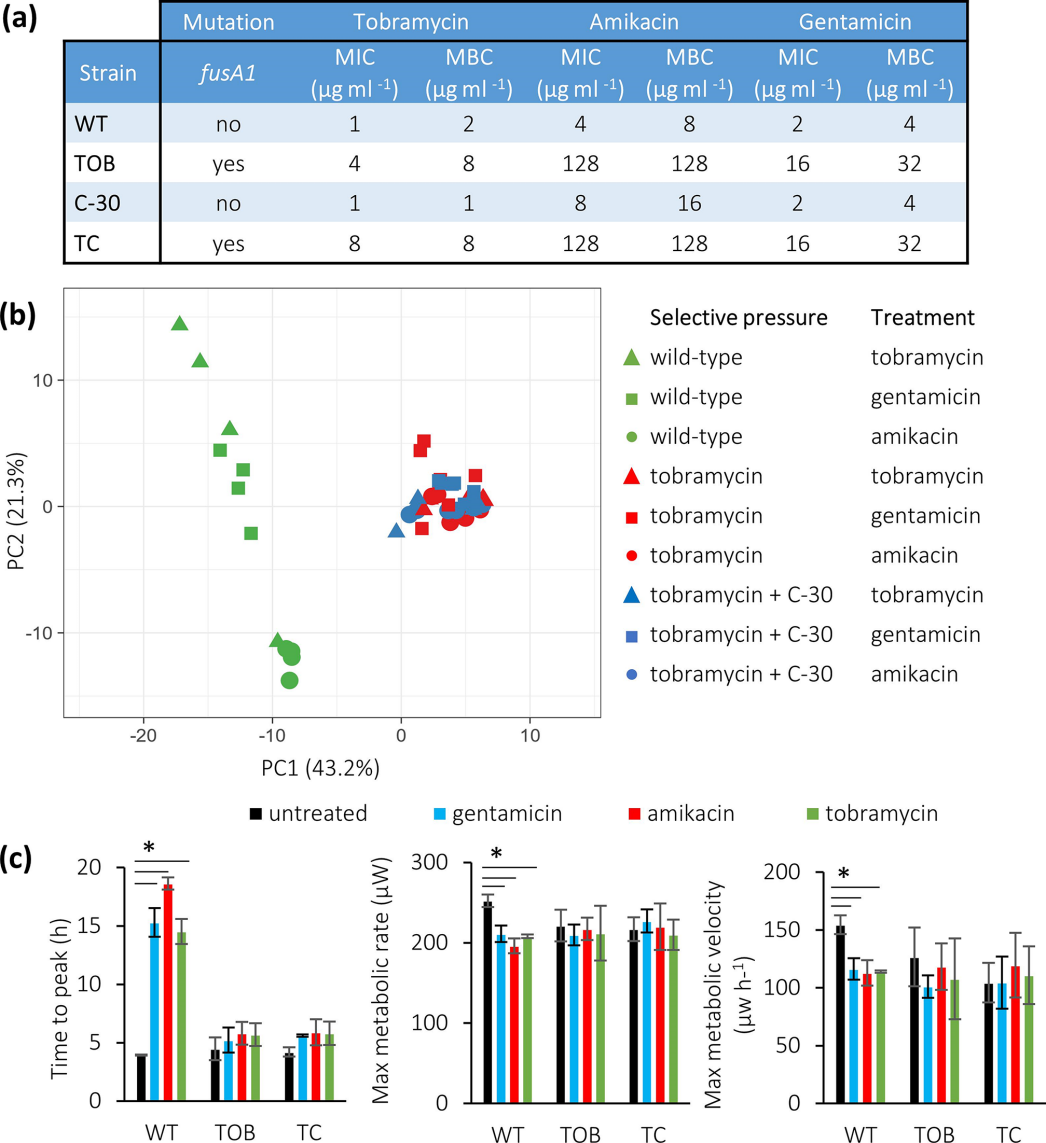
(a) Median MIC and MBC of tobramycin, amikacin and gentamicin for the WT and evolved strains. (b) PCA plot of thermograms of the WT and evolved strains treated with tobramycin (0.25 µg ml^−1^), amikacin (1 µg ml^−1^) and gentamicin (1 µg ml^−1^). (c) Average time to peak (left), maximum metabolic rate (centre) and maximum metabolic velocity (right). WT: wild-type, TOB: lineage evolved in the presence of tobramycin, TC: lineage evolved in the presence of the combination of tobramycin and C-30. Data shown are average, error bars indicate standard deviations (*n*=3). *, Significantly different from the untreated control (*P*<0.05).

The MIC and MBC of the aminoglycoside antibiotics was increased (4- to 32-fold, Fig. 3a) for all strains that were exposed to tobramycin (either alone or combined with C-30), which is likely related to the mutation in *fusA1*, present in all lineages. In the text and figures we refer to the median values across multiple lineages evolved in the same conditions; MIC for individual lineages can be found in Table S1.

Thermograms obtained with lineages that were evolved in the presence of tobramycin, and were treated with amikacin, gentamicin or tobramycin, were similar to each other and clustered together. Thermograms obtained with the WT strain clustered according to the treatment and were different from the thermograms of the evolved strains (Fig. 3b). This clustering is in line with significant differences in time to maximum metabolic activity, maximum metabolic activity and maximum metabolic velocity (Fig. 3c).

### Response to ciprofloxacin treatment

**Fig. 4.**
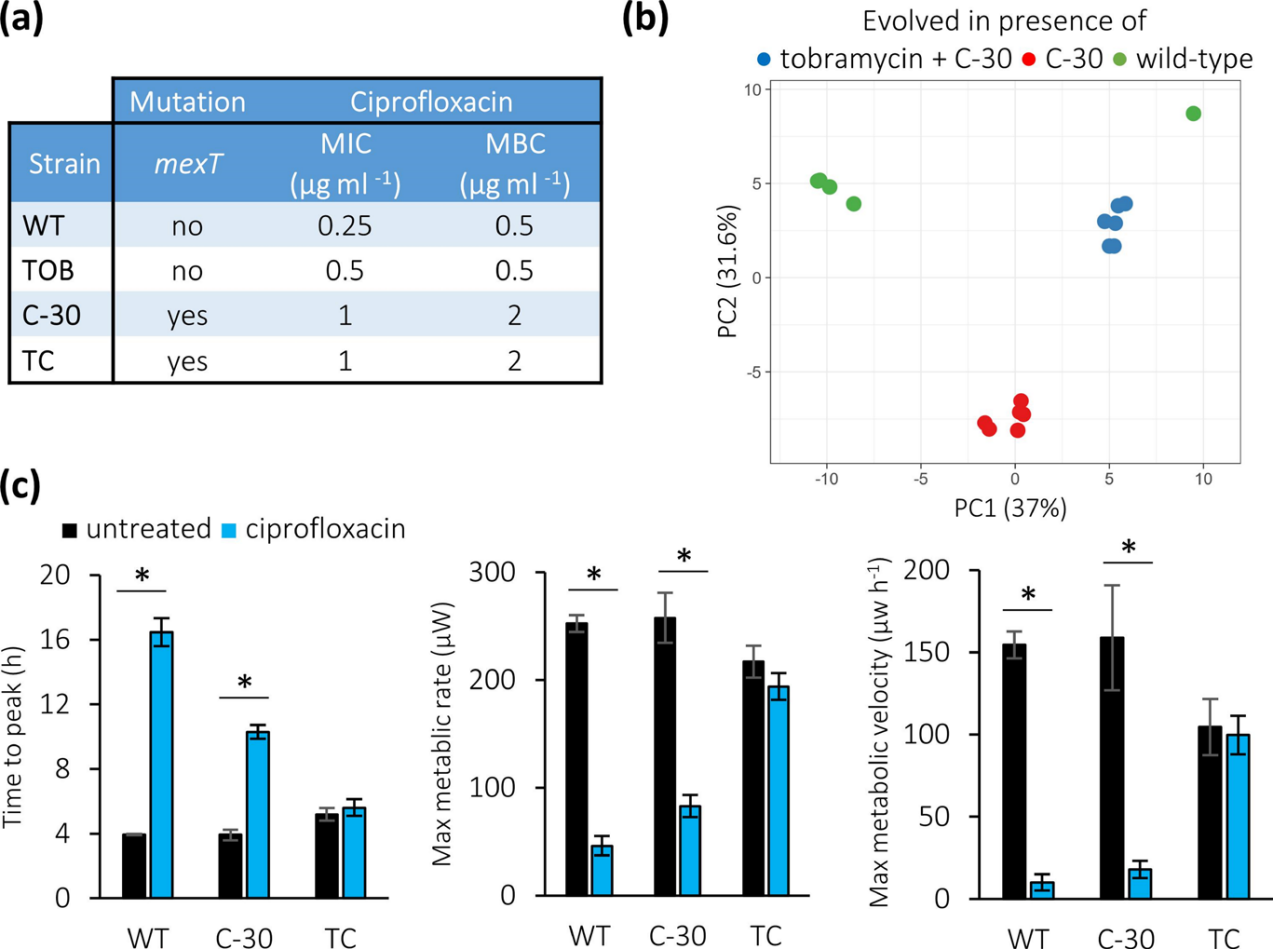
(a) Median MIC and MBC of ciprofloxacin. (b) PCA plot of thermograms of the WT and evolved strains treated with ciprofloxacin. (c) Average time to peak (left), maximum metabolic rate (centre) and maximum metabolic velocity (right) of the WT and evolved *P. aeruginosa* lineages in the presence (blue) and absence (black) of treatment with ciprofloxacin (0.125 µg ml^−1^). WT: wild type, C-30: lineage evolved in the presence of C-30, TC: lineage evolved in the presence of the combination of tobramycin and C-30. Data shown are average, error bars indicate standard deviations (*n*=3). *, Significantly different from the untreated control (*P*<0.05).

The MIC and MBC of ciprofloxacin for all lineages with the 8 bp deletion in *mexT* (all lineages evolved in the presence of C-30, either alone or combined with tobramycin) were increased fourfold compared to the values observed for the WT *P. aeruginosa* PAO1, while the MIC for the evolved strains that were exposed to tobramycin alone (which do not have the deletion in *mexT*) was unchanged (Fig. 4a). The thermograms of the WT strain could easily be distinguished from those of the strains evolved in presence of C-30 (alone and together with tobramycin) (Fig. 4b). In addition, thermograms obtained with strains evolved in the presence of C-30 alone were different from those obtained with strains evolved in the presence of the combination C-30 and tobramycin, despite the fact that ciprofloxacin MIC and MBC were identical for these strains. The time to maximum metabolic activity of both the WT *P. aeruginosa* and strains evolved in the presence of C-30 alone was significantly increased during treatment with ciprofloxacin, while the maximum metabolic rate and velocity were significantly decreased. For the strains evolved in the presence of the combination, the time to maximum metabolic activity, the maximum metabolic rate and the maximum velocity in the presence of ciprofloxacin was unaltered (Fig. 4c). Our results show that the strains evolved in the presence of C-30 (either alone or combined with tobramycin) have a decreased susceptibility to ciprofloxacin, likely linked to the deletion in *mexT*.

### Response to chloramphenicol treatment

**Fig. 5.**
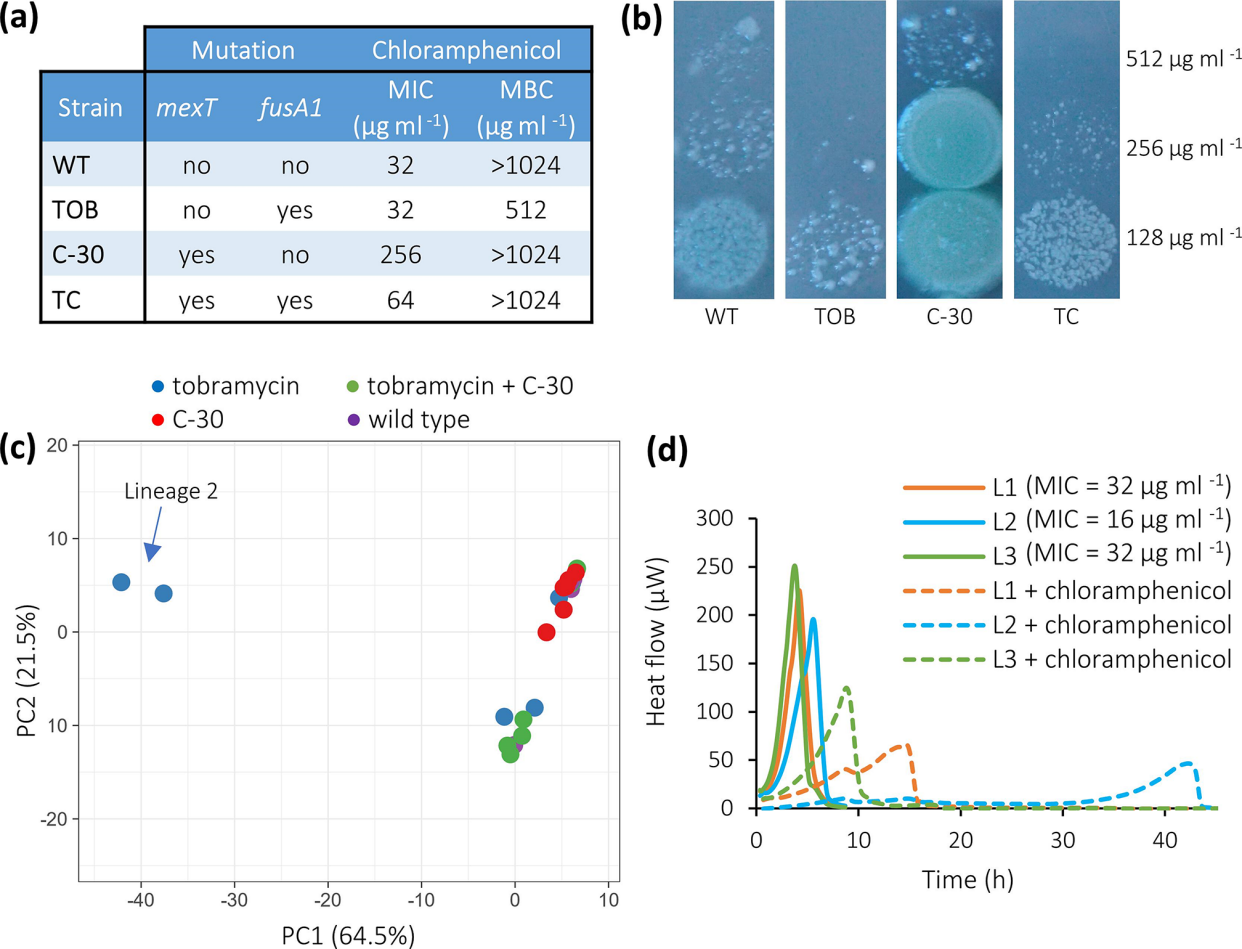
(a) Median MIC and MBC of chloramphenicol for the wild-type *P. aeruginosa* (WT) and for the strains evolved in presence of tobramycin (TOB), C-30 (C-30) or tobramycin combined with C-30 (TC). (b) Growth on LB agar of the WT and evolved *P. aeruginosa* after MIC determination (bacteria belonging to lineage 1). (c) PCA plot of thermograms of the WT and strains evolved in the presence of tobramycin, C-30 and a combination of tobramycin and C-30 after treatment with chloramphenicol (16 µg ml^−1^). (d) Thermograms of the three lineages (L1, orange; L2, blue; and L3, green) that were experimentally evolved in the presence of tobramycin, treated with chloramphenicol (dotted lines) or untreated (full lines), and median MIC of chloramphenicol for the three independent lineages.

The MIC of chloramphenicol for the strains evolved in the presence of C-30 alone was drastically increased (from 32 to 256 µg ml^−1^). Surprisingly, the MIC only increased twofold for the strains evolved in the presence of the combination C-30 +tobramycin (Fig. 5a). Besides mutations in *mexT*, strains evolved in this condition also have a mutation in *fusA1* (Fig. 5a). It was previously observed that *fusA1* mutations resulted in an increased susceptibility of *Salmonella* to chloramphenicol [57] and in *E. coli* collateral sensitivity to chloramphenicol was found to be due to mutations in *fusA1* [58]. It seems reasonable to speculate that the observed *fusA1* mutation also increases their susceptibility to chloramphenicol, counteracting the effect of the 8 bp deletion in *mexT* on chloramphenicol susceptibility. In addition, we observed that the MBC of chloramphenicol for strains evolved in the presence of tobramycin alone was 512 µg ml^−1^, which is lower than the MBC for the other evolved strains and the WT (> 1024 µg ml^−1^) (Fig. 5a). Plating of the culture after 24 h of exposure to various concentrations of chloramphenicol (Fig. 5b) confirmed that the WT strain is more susceptible than strains evolved in the presence of C-30 alone, possibly due to the deletion in *mexT* that is observed in the latter. Likewise, strains evolved in the presence of tobramycin alone are more susceptible to chloramphenicol than the WT strain (Fig. 5b), which might be due to the mutation in *fusA1*. Finally, growth of the WT *P. aeruginosa* was similar to the growth of the strains that were evolved in the presence of the combination of tobramycin and C-30 (Fig. 5b).

Thermograms derived from strains evolved in the presence of C-30 and C-30 +tobramycin (both carrying the deletion in *mexT*) were clearly separated from each other in the PCA plot (Fig. 5c). Whether this was due to the presence of a mutation in *fusA1* in strains evolved in the presence of the combination but not in the strains evolved in presence of C-30 alone remains to be determined. The thermograms derived from lineage 2 clustered separately from those of lineages 1 and 3 (Fig. 5c) and showed an increased susceptibility to chloramphenicol (MIC of 16 µg ml^−1^ vs. 32 µg ml^−1^ for lineages 1 and 3). The metabolic activity of lineage 2 was indeed affected much more by treatment with chloramphenicol than that of lineages 1 and 3 (Fig. 5d). Exposure to chloramphenicol had a large impact on the heat flow of the evolved and WT strains (Fig. 2), and consequently, the time to maximum metabolic activity, the maximum metabolic activity and maximum metabolic velocity of all the evolved strains and the WT *P. aeruginosa* was significantly changed after treatment with chloramphenicol (Fig. S3).

### Evolved lineages with an 8 bp deletion in *mexT* show decreased susceptibility to C-30

It has been proposed that QSI are less likely to induce selective pressure and consequently, resistance towards them would develop slower and less frequent [18]. However, *P. aeruginosa* isolates that have repeatedly been exposed to C-30 quickly acquired a mutation in *mexT* [30]. This suggests that this mutation is beneficial to the bacteria in the presence of C-30, and that C-30 exerts a selective pressure on *P. aeruginosa *, which may be related or unrelated to its QS inhibitory activity. Indeed, QSIs that not only inhibit QS, but also inhibit other essential pathways have been described, and these could potentially also impact growth and/or metabolism [22].

**Fig. 6.**
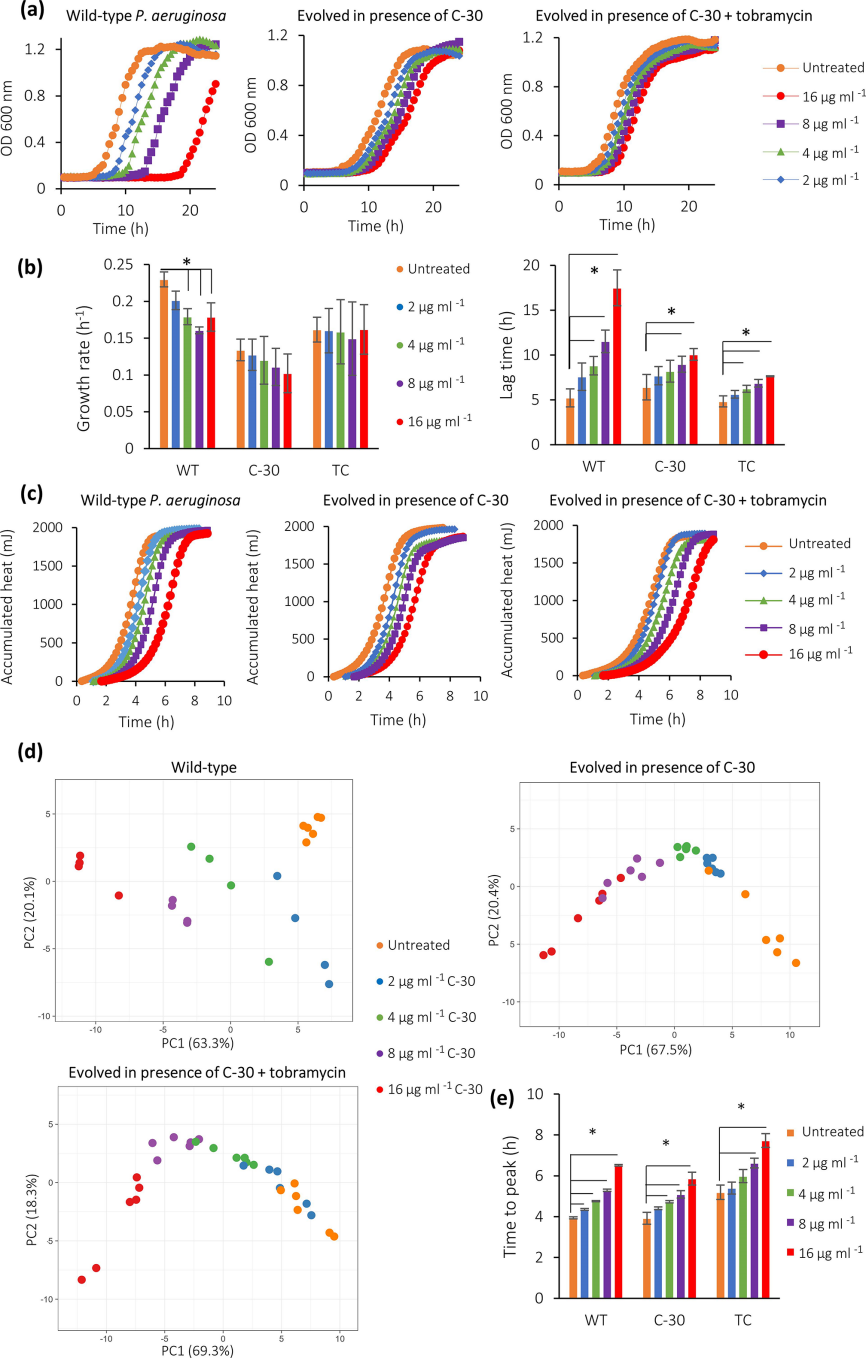
(a) Growth curves, (b) growth rate and lag time, (c) accumulated heat, (d) PCA plot of thermograms and (e) time to maximum metabolic activity, of *P. aeruginosa* PAO1 wild-type (WT) and the experimentally evolved lineages repeatedly exposed to C-30 (C-30) and a combination of C-30 and tobramycin (TC), who both have a mutation in *mexT*, in the absence and presence of 2, 4, 8 and 16 µg ml^−1^ of C-30. Data shown are average, error bars indicate standard deviations (*n*=3). *, Significantly different from the untreated control (*P*<0.05).

We investigated the effect of C-30 on the growth of *P. aeruginosa *, and determined whether the mutation in *mexT* had an impact on the susceptibility of *P. aeruginosa* to C-30 (Fig. 6a). Exposure of WT *P. aeruginosa* PAO1 to increasing concentrations of C-30 significantly increased the duration of the lag phase, while the duration of the lag phase of the evolved lineages with an 8 bp deletion in *mexT* was much less affected by C-30 (Fig. 6b). The growth rate during exponential phase of the wild-type *P. aeruginosa* was significantly affected by C-30 (approx. 20 % decreased after exposure to the highest dose of C-30), while the growth rate of the evolved strains was not significantly affected by C-30 (Fig. 6b). C-30 affected the total accumulated heat of both WT and evolved strains in a concentration-dependent way (Fig. 6c). Thermograms derived from the WT strain clustered according to the different concentrations of C-30 (Fig. 6d). Thermograms derived from strains that were evolved in the presence of C-30 (either alone or combined with tobramycin) could not always be distinguished from each other, although the thermograms derived from untreated strains and from strains treated with 16 µg ml^–1^ C-30 clustered separately in the PCA plot. The time to maximum metabolic activity of the WT was significantly increased after exposure to C-30 (Fig. 6e), while the time to maximum metabolic activity of the strains evolved in the presence of C-30 alone and the combination of C-30 and tobramycin, was only significantly increased after treatment with 8 and 16 µg ml^–1^ C-30.

The maximum metabolic rate and maximum metabolic velocity of the WT and evolved strains were not affected by exposure to C-30 (Fig. S4). These data indicate that the QSI C-30 delays both the start of growth and the metabolic activity of *P. aeruginosa *, but only slightly impacts on the growth rate of the WT, and has no effect on the metabolic velocity during the exponential growth phase. In addition, strains experimentally evolved in presence of C-30 (with an 8 bp deletion in *mexT*) are less susceptible to its effect on growth and metabolism.

### Expression of *mexE* is induced by exposure to C-30 and is significantly increased in lineages that have a deletion in *mexT*

**Fig. 7.**
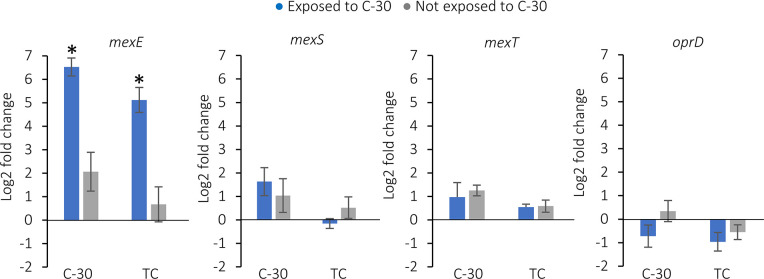
Log2 fold change of *mexE, mexS, mexT* and *oprD* expression in the strains evolved in presence of C-30 or the combination of C-30 and tobramycin (TC), compared to the expression in WT *P. aeruginosa* PAO1, with and without exposure to C-30. Data shown are average, error bars represent standard deviations (*n*=3). *, Significant change in expression compared to the WT *P. aeruginosa* PAO1 (*P*<0.0001).

The effect of the 8 bp deletion in *mexT* on the expression of *mexEF-oprN* was determined using RT-qPCR (Fig. 7). In the absence of C-30, the expression of *mexE* in the evolved lineages was not significantly different from the expression in the WT. When the bacteria were exposed to C-30 30 min prior to RNA extraction, the expression of *mexE* was significantly increased in the strains that have the 8 bp deletion in *mexT* (*P*<0.0001). Genome wide expression profiling of *P. aeruginosa* PAO1 also showed an increased expression of *mexEF* after exposure to C-30 [21]. These data support the hypothesis that C-30 is actively exported by MexEF-OprN. Furthermore, RT-qPCR confirms that the expression of *mexEF-oprN* is upregulated in the *mexT* mutants, which likely explains the decreased antimicrobial susceptibility of the evolved lineages to ciprofloxacin (Fig. 4a) and chloramphenicol (Fig. 5a). When C-30 was added at the start of the experiment (i.e. about 7 h prior to the RNA-extraction) the expression of *mexT* in the evolved lineages was not significantly changed compared to the expression in the WT (data not shown). This result is in line with the changes that were observed in the growth of *P. aeruginosa* in the presence of C-30 (Fig. 6a) and indicates that C-30 affects *P. aeruginosa* after initial exposure only, but over time *P. aeruginosa* adapts and becomes less affected. As the growth of *mexT* mutants was less affected by C-30 than the WT strain, due to an increased efflux of C-30, it is likely that QS in *mexT* mutants will also be less affected by C-30 and this might contribute to the decreased antimicrobial activity of the tobramycin/C-30 combination against the evolved bacteria.

The expression of *mexS* and *mexT*, the genes encoding the two transcriptional regulators of MexEF-OprN, was not different in the evolved lineages compared to the WT *P. aeruginosa *, even after exposure to C-30. While MexT is thought to co-regulate the expression of *mexS* [11] the 8 bp deletion in *mexT* that restores its function [30] did not have an impact on the expression of *mexS* in our study.

The expression of *oprD* (Fig. 7) in the evolved lineages with the deletion in *mexT* was unchanged compared to WT, which is in contrast to results obtained in previous studies, in which reduced *oprD* expression was attributed to mutations in *mexT* [12, 59]. The unchanged *oprD* expression is in line with the susceptibility of the evolved lineages to imipenem, which was unchanged compared to WT (MIC of imipenem was 4 µg ml^−1^ for all strains investigated) [13].

### *In vitro* production of virulence factors is not affected by repeated exposure to C-30

**Fig. 8.**
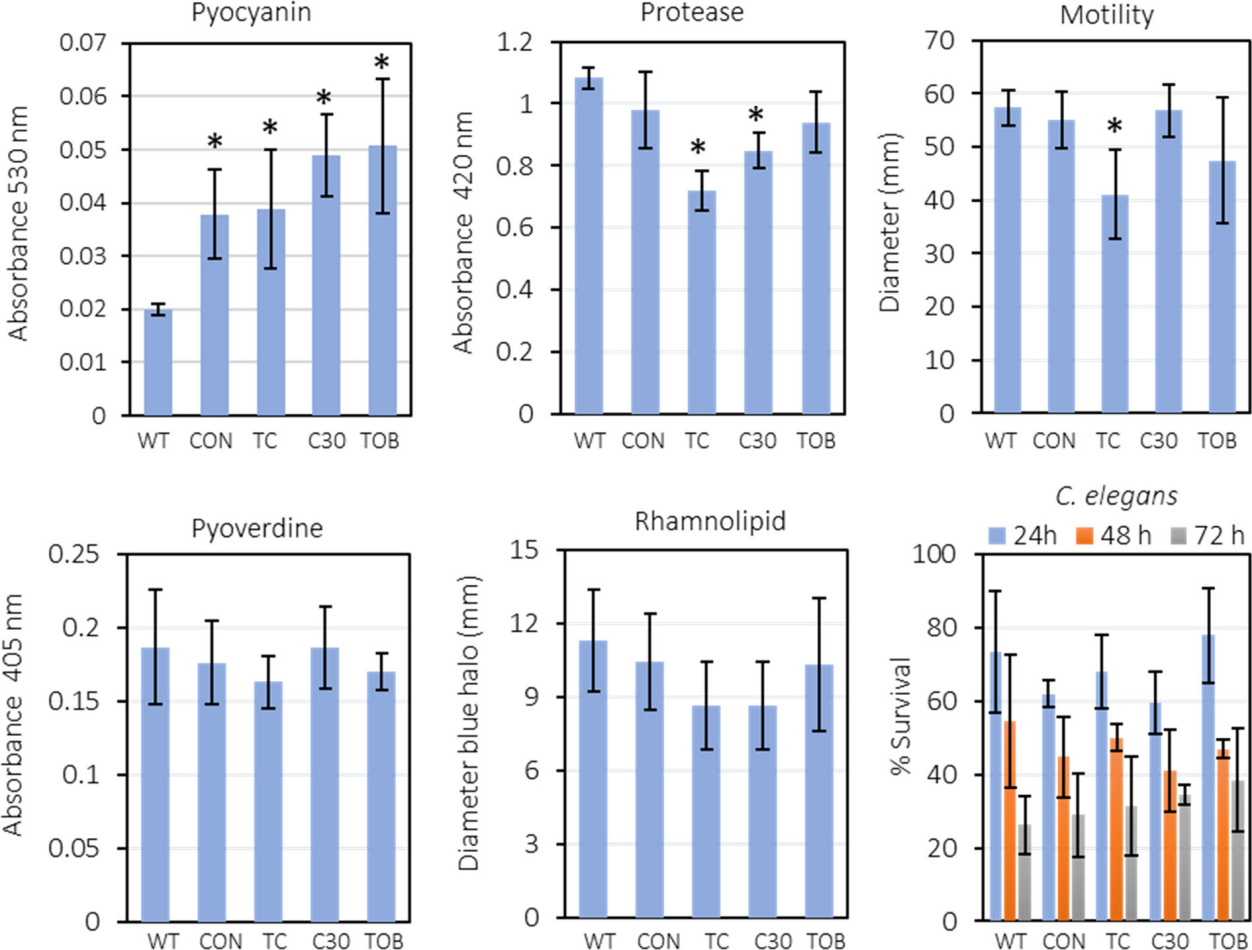
The average pyocyanin, protease, pyoverdine and rhamnolipid production, swarming motility and virulence in a *C. elegans* infection model, of the wild-type *P. aeruginosa* (WT), of strains experimentally evolved in the presence of tobramycin (TOB), C-30 (c30), or a combination of both (TC), and of an untreated evolved control (CON). Data shown are average, error bars represent standard deviations (*n*=9). * Significantly different from the WT *P. aeruginosa* (*P*<0.05).

We investigated the *in vitro* production of virulence factors including pyocyanin, pyoverdine, proteases and rhamnolipids as well as the swarming motility of the WT and evolved strains (Fig. 8). Because many virulence factors are regulated via QS [60], repeated exposure to a QSI might influence the virulence of the experimentally evolved lineages. Furthermore, previous research indicated that MexEF-OprN overexpression mutants with a mutation in *mexT* would produce less virulence factors, such as pyocyanin, elastase and rhamnolipids [61, 62].

The pyocyanin production of all evolved lineages (including an untreated control) was increased compared to the WT *P. aeruginosa* PAO1. This suggests that repeated growth in SCFM2 leads to increased pyocyanin production, but that this effect is unrelated to repeated exposure to the QSI or to the mutation in *mexT*. The protease activity of the lineages evolved in the presence of C-30 or C-30 and tobramycin (i.e. all lineages that have the *mexT* mutation) was significantly decreased compared to the WT. In addition, the motility of *P. aeruginosa* experimentally evolved in the presence of C-30 and tobramycin was significantly decreased (approx. 30%). No differences in pyoverdine and rhamnolipid production were observed. Overall, we can conclude that repeated exposure to C-30 did not result in changes in the production of virulence factors.

### Virulence and effect of antimicrobial treatment in *C. elegans* and in a mouse peritonitis infection model

The virulence of the WT and experimentally evolved *P. aeruginosa* was first assessed in an *in vivo C. elegans* model. After 3 days of infection, the number of surviving nematodes was approx. 30 % for all strains investigated, suggesting that virulence of *P. aeruginosa* did not change after experimental evolution (Fig. 8).

**Fig. 9.**
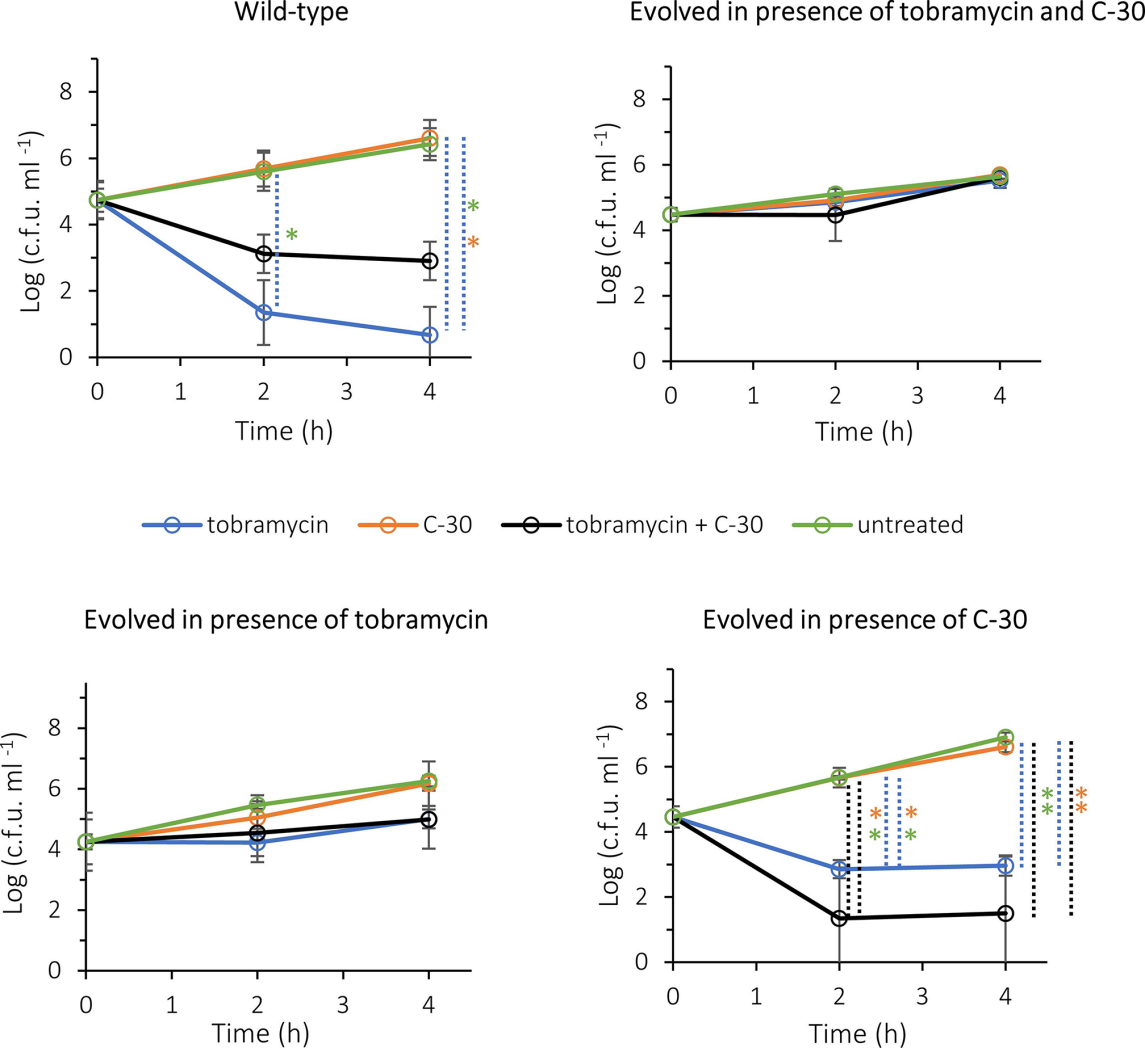
Number of culturable bacteria in the blood of mice infected with WT and evolved *P. aeruginosa* (lineage 3), 2 and 4 h after treatment with tobramycin, C-30, or a combination of both, and of infected, untreated control mice. Data shown are average, error bars indicate standard deviations (*n*=4) *, Significant difference in the number of c.f.u. after treatment (*P*<0.05).

Subsequently, virulence and the effect of an antimicrobial treatment *in vivo* were evaluated in a mouse peritonitis infection model [48]. To this end mice were infected intraperitoneally with the WT or evolved strains and 1 h post-infection tobramycin, C-30 or a combination of both was administered subcutaneously; the number of culturable bacteria in blood (Fig. 9) and peritoneal fluid (Fig. S5) was determined 2 and 4 h after treatment. The number of bacteria recovered from the blood from infected and untreated mice after 5 h of infection with the WT and evolved strains, was about 10^6^ c.f.u. ml^−1^ in all cases (Fig. 9). In line with this, no differences could be observed in the clinical appearance of the mice, animals in all groups were scored as ‘clearly affected’, which means they only move when pushed, have spiky fur, half-closed eyes and a curved back.

Treatment with tobramycin alone significantly reduced the number of culturable WT *P. aeruginosa* in the blood of infected mice; addition of C-30 did not further reduce this number. For bacteria evolved in the presence of tobramycin (either alone or combined with C-30), the number of c.f.u. in the blood of infected mice was not affected by treatment with tobramycin, while for the bacteria evolved in presence of C-30 alone and for the evolved control bacteria (Fig. S6), there was a significant decrease after treatment with tobramycin alone or tobramycin combined with C-30. C-30 alone had no effect on the number of culturable bacteria after infection with the WT and evolved strains. The effect of the treatments on the number of surviving bacteria in the peritoneal fluid was in line with the effect on the number of bacteria in the blood, with one exception (the number of surviving bacteria evolved in the presence of the combination surviving exposure to tobramycin *in vivo* was decreased in peritoneal fluid alone). These results indicate that resistance to tobramycin of the strains that were evolved in its presence *in vitro*, also result in a decreased tobramycin susceptibility *in vivo* for tobramycin (alone and combined with C-30).

## Conclusion

We have previously shown that that experimental evolution of *P. aeruginosa* PAO1 in the presence of of tobramycin and C-30 rapidly leads to reduced susceptibility [15] and here we show that this reduced susceptibility carries a fitness cost in the absence of these compounds. Evolution in the presence of C-30, tobramycin and their combination leads to reduced susceptibility to MexEF-OprN substrates and aminoglycoside antibiotics, due to mutations in *mexT* and *fusA1,* respectively. Exposure to C-30 resulted in a reduced susceptibility, linked to a mutation in *mexT*. Microcalorimetry revealed that changes in susceptibility were in many cases linked to changes in metabolic activity. Repeated exposure to C-30 had no effect on the production of virulence factors and the results from *in vivo* experiments indicate that there are no trade-offs between reduced susceptibility and virulence. Finally, the reduced susceptibility observed for some strains *in vitro* were also observed *in vivo*.

In conclusion, our data indicate that *P. aeruginosa* is able to rapidly adapt during repeated exposure to the aminoglycoside antibiotic, tobramycin, and the QSI, C-30 (alone or combined). These adaptations have an impact on the antimicrobial susceptibility of *P. aeruginosa *, not only towards compounds that were included in the initial therapy but also towards antimicrobials belonging to different classes. In addition, a mechanism of resistance against furanone C-30 was detected, which confirms that treatment of *P. aeruginosa* with QSI can induce development of resistance, similar to when *P. aeruginosa* is treated with conventional antibiotics.

## Supplementary Data

Supplementary material 1
