# Supplementary material for: Adaptation of Pseudomonas aeruginosa biofilms to tobramycin and the quorum sensing inhibitor C-30 during experimental evolution requires multiple genotypic and phenotypic changes
Source: Microbiology (Reading). 2023 Jan 18;169(1):001278. doi: 10.1099/mic.0.001278 (PMC9993117; doi:10.1099/mic.0.001278)
Supplement: Supplementary material 1 [file mic-169-1278-s001.pdf]

SUPPLEMENTARY TABLES AND FIGURES

Table S1: Median MIC (µg/ml) for each individual lineage (L1, L2 and L3) of ciprofloxacin (CIP), imipenem (IPM), chloramphenicol (CHL), tobramycin (TOB), amikacin (AMK) and gentamycin (GEN) (n = 3).

Strains evolved in presence of C-30 + tobramycin						
Strain	CIP	IPM	CHL	TOB	AMK	GEN
WT (Cycle 0)	0.25	4	32	1	4	2
L1 cycle 16	1	2	64	4	64	16
L2 cycle 16	1	4	32	8	128	16
L3 cycle 16	0.5	8	64	8	128	16
Strains evolved in presence of tobramycin						
Strain	CIP	IPM	CHL	TOB	AMK	GEN
WT (Cycle 0)	0.25	4	32	1	4	2
L1 cycle 16	0.5	4	32	8	128	16
L2 cycle 16	0.5	2	16	4	32	16
L3 cycle 16	0.5	8	32	8	128	16
Strains evolved in presence of furanone C-30						
Strain	CIP	IPM	CHL	TOB	AMK	GEN
WT (Cycle 0)	0.25	4	32	1	4	2
L1 cycle 16	1	2	128	1	8	2
L2 cycle 16	1	4	256	1	8	2
L3 cycle 16	0.5	4	256	1	8	2

Table S2: Genes that were mutated after experimental evolution

Evolved in presence of	Lineage	Mutated genes
Tobramycin	1	*fusA1, parS*
	2	*fusA1, hasS*
	3	*fusA1, parS, ptsP*
Furanone C-30	1	*mexT, dipA, rbdA, rpoS*
	2	*mexT*
	3	*mexT, htpG, pirR, pirS*
Tobramycin + furanone C-30	1	*fusA1, mexT, rne, rpsE*
	2	*fusA1, mexT, htpG*
	3	*fusA1, mexT, parS, rne*

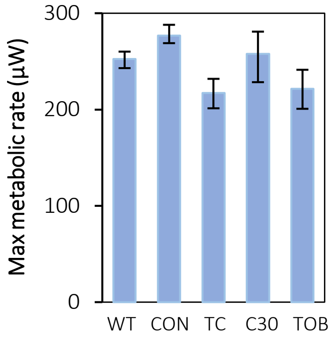

Figure S1: Average maximum metabolic activity of wild type (WT), evolved control (CON) and strains evolved in presence of tobramycin and C-30 (TC), C-30 or tobramycin (TOB). Error bars indicate standard deviations (n = 3). No significant differences could be detected between the wild type and evolved strains.

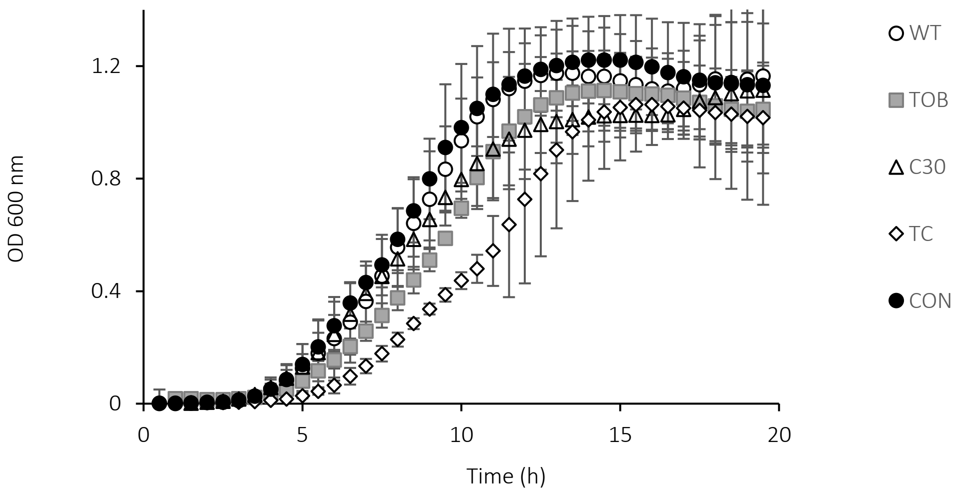

Figure S2: Growth curve of the different *P. aeruginosa* strains investigated. WT: wild type, CON: lineage evolved in the absence of antimicrobial treatment, C30: lineage evolved in the presence of C-30, TC: lineage evolved in the presence of the combination of tobramycin and C-30, TOB: lineage evolved in the presence of tobramycin. Data shown are average, error bars indicate standard deviations (n = 3).

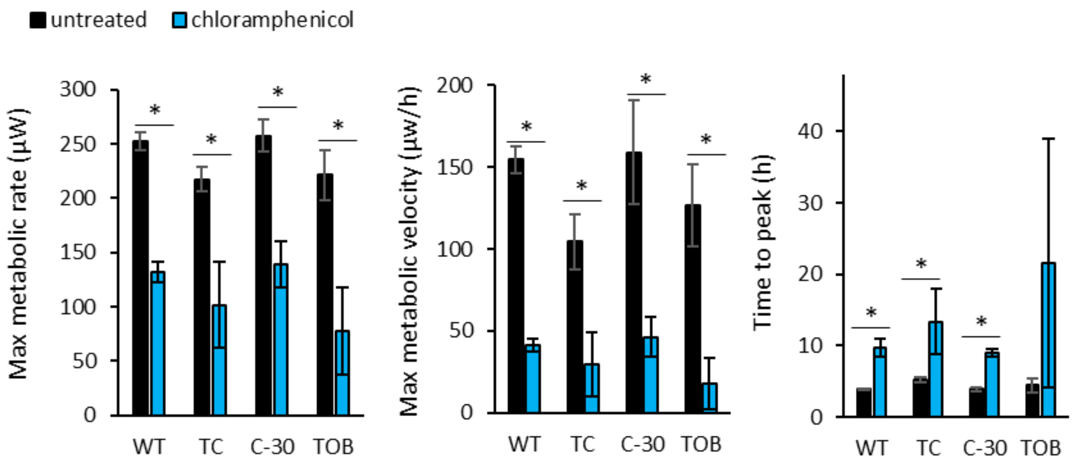

Figure S3: Maximum metabolic activity (left), maximum metabolic velocity (center) and time to peak (right) of wild type *P. aeruginosa* (WT) and strains evolved in presence of tobramycin and C-30 (TC), C-30 (C-30) or tobramycin (TOB) with (blue) and without (black) treatment with chloramphenicol. Data shown are average, error bars indicate standard deviations (n = 3). *****, Significantly different from the untreated strain ($p < 0.05$).

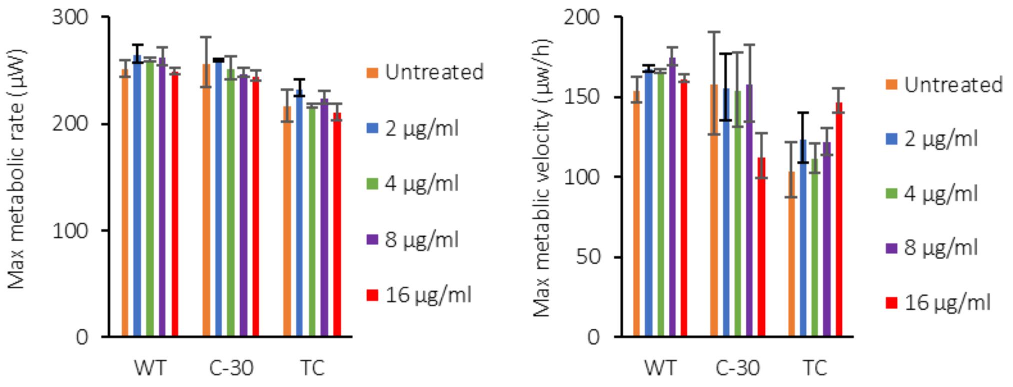

Figure S4: Maximum metabolic activity (left) and maximum metabolic velocity (right) of wild type *P. aeruginosa* (WT) and strains evolved in presence of C-30 (C-30) and a combination of C-30 and tobramycin (TC), untreated and treated with 2, 4, 8 and 16 µg/ml of C-30. Data shown are average, error bars indicate standard deviations (n = 3). No significant differences could be observed between the untreated and treated bacteria.

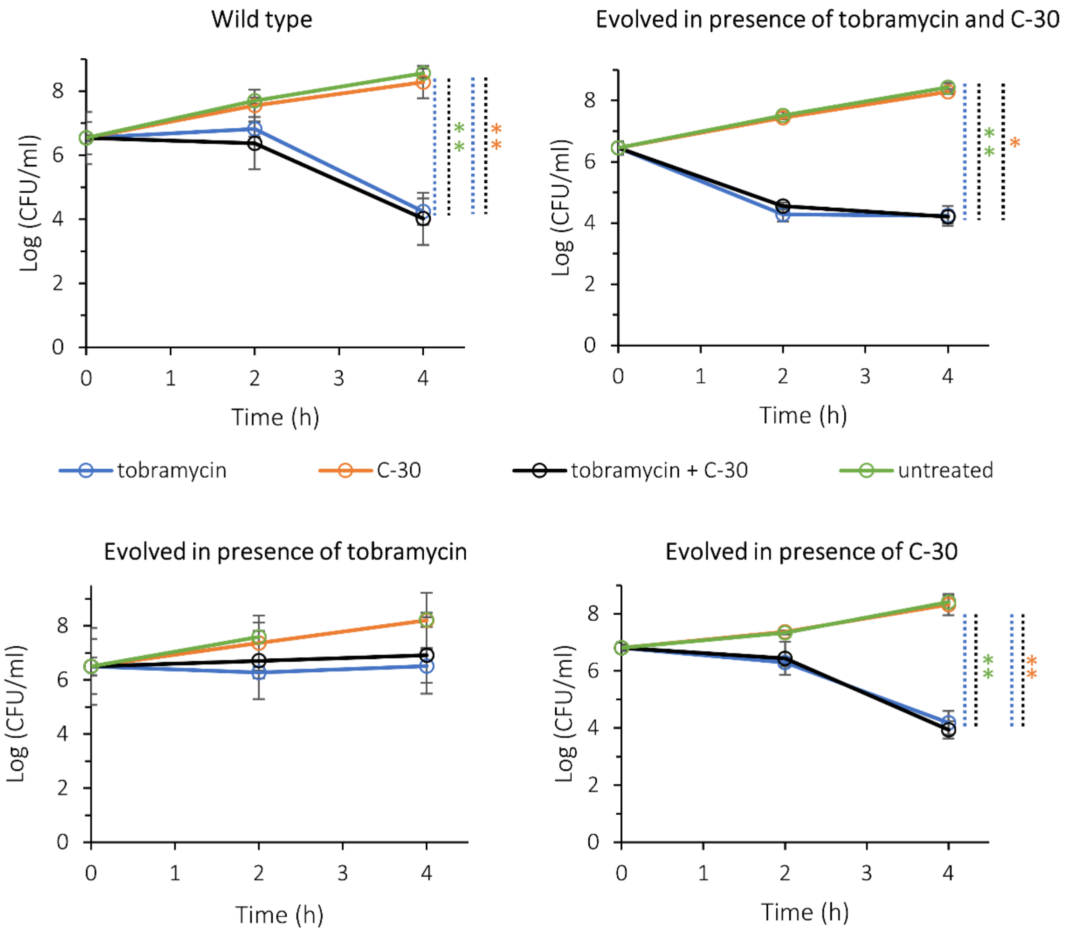

Figure S5: Average number of bacteria in the peritoneal fluid of mice infected with wild type and evolved *P. aeruginosa* (lineage 3), 2 and 4 h after treatment with tobramycin, C-30, or a combination of both, and of infected, untreated control mice. Error bars indicate standard deviations (n = 4) * Significant difference in the number of CFU, 4 h after treatment ($p < 0.05$).

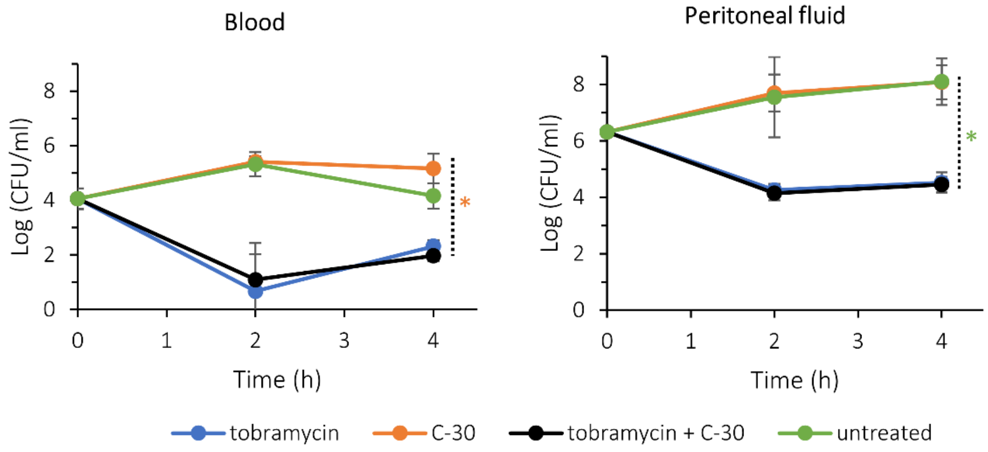

Figure S6: Average number of bacteria in the blood and peritoneal fluid after infection with the evolved control, 2 and 4 h after treatment with tobramycin, C-30, or a combination of both, and of infected, untreated control mice. Error bars indicate standard deviations (n = 4) * Significant difference in the number of CFU, 4 h after treatment ($p < 0.05$).
